# Effects of Adding Astragali Radix and Inulae Radix on Fermentation Quality, Nutrient Preservation, and Microbial Community in Barley Silage

**DOI:** 10.3390/microorganisms13122822

**Published:** 2025-12-11

**Authors:** Ying Yun, Ying Ying, Juanjuan Sun, Jinmei Zhao, Wenxi Wang, Boyang Kang

**Affiliations:** 1 Institute of Grassland Research, Chinese Academy of Agricultural Science, Hohhot 010010, China; yunying@caas.cn (Y.Y.); sunjuanjuan@caas.cn (J.S.); zhaojinmei@caas.cn (J.Z.); wangwenxi@caas.cn (W.W.); 2 Inner Mongolia Agricultural and Livestock Product Quality and Safety Center, Hohhot 010010, China; 18747980714@163.com

**Keywords:** barley silage, *Astragalus membranaceus* L., *Inula helenium* L., fermentation and nutrient quality, microbial community

## Abstract

Chinese herbal medicine (CHM) residues represent a promising and sustainable category of silage additives, with the potential to modulate fermentation and enhance nutrient preservation. This study investigated the effects of two CHMs, *Astragalus membranaceus* L. (Astragali Radix, AR) and *Inula helenium* L. (Inulae Radix, IR), on the fermentation profile, nutritional composition, and bacterial community structure in barley silage. The forage was ensiled without additive (control, CK), or with 1% or 2% (*w*/*w*) of AR or IR for 75 days. The results showed that all additive treatments significantly improved fermentation quality, as evidenced by lower pH and reduced ammonia-nitrogen (NH_3_-N) content compared to CK. The 2% IR treatment was most effective in promoting homolactic fermentation, yielding the highest lactic acid content and lactic acid-to-acetic acid ratio. Nutritionally, additives significantly increased dry matter, starch, and water-soluble carbohydrates, while decreasing neutral and acid detergent fiber contents. High-throughput sequencing of the 16S rRNA gene revealed that both herbal additives profoundly reshaped the microbial community. They suppressed undesirable bacteria and significantly enriched beneficial *Lactobacillus* species. Principal component analysis confirmed a distinct separation in microbial community structure between control and treated silages. These findings underscore the potential of these herbal residues as natural modulators of the silage microbiome for improved forage conservation.

## 1. Introduction

Barley (*Hordeum vulgare* L.) is a cheap source that prefers to germinate and obtain easier than corn and other forage. In the study of forage preservation, ensiling plays a vital role in maintaining the nutritional quality of moist forage crops [1]. It has been extensively utilized in livestock farming to ensure an adequate supply of high-quality feed throughout the year [2,3]. Barley forage contains a high amount of water-soluble carbohydrates providing substrate for the production of lactate to ensure preservation [4]. Depending on the maturity stage at harvest, barley silage exhibits a significant variation in cell wall content, with neutral detergent fiber (NDF) ranging from below 40% to over 60% [5]. Previous reports indicated that barley silage exhibited higher levels of crude protein (CP) and enhanced digestibility of dry matter (DM) compared to other evaluated small grain cereal silage. Substituting corn silage with barley silage, either partially or entirely, in dairy cows’ diets had no adverse effects on animal performance, milk quality characteristics, cheese quality, or yield [6].

The application of additives during ensiling is a well-established method to enhance silage quality [7,8,9,10]. Beyond conventional options like microbial inoculants, Chinese herbal medicines (CHMs) have recently emerged as promising natural alternatives [11]. China, as a major consumer of herbal medicine resources, is experiencing rapid growth in the production of herbal residues driven by the advancement of its traditional Chinese medicine industry. The annual discharge of these residues has reached 60–70 million tons and continues to increase, posing potential risks such as soil and water pollution and disruption of the ecological balance [12,13]. Herbal residues contain biologically active compounds such as alkaloids, polysaccharides, and terpenes [14], which provide nutritional benefits and exert regulatory effects in biological systems. Wu et al. [11] reported adding eleven types of herbal medicine residues to corn straw silage. The treatments raised lactic acid to 34.3–45.3 g kg^−1^ DM, cut neutral/acid-detergent fiber by up to 48.3/32.7 g kg^−1^ DM, reduced NH_3_-N from 3.46 to 0.42–1.68 g kg^−1^ DM, and boosted *Lactiplantibacillus plantarum* from 3.6% to 30–65% of the community while suppressing *Bacillus licheniformis*. Li et al. [15] showed that incorporating thirty-six herbal residues at 2.5% of alfalfa fresh weight lowered pH to 4.37–4.71, cut NH_3_-N/TN from 10.2% down to 2.46%, and lifted lactic acid to 105.5 g kg^−1^ DM. High-throughput sequencing revealed that the residues enriched *Lactobacillus* to 61–78% while suppressing *Enterobacter* and *Clostridium*, enhanced carbohydrate metabolism, and reduced antibiotic-resistance genes. Ni et al. [16] reported that incorporating 2.5% *Astragalus* (AS) and 3% *Hawthorn* (HN) residues into alfalfa silage for 60 days cut butyric acid to 1.31 g kg^−1^ DM and NH_3_-N from 123 to 11.9 g kg^−1^ TN, while qPCR quantification showed parallel reductions in *Clostridium*, *Enterobacter,* and fungal copy numbers, demonstrating that the AS + HN combination synergistically suppresses spoilage microbes and upgrades silage quality and resource utilization. Collectively, these studies demonstrate that Chinese herbal medicine additives can effectively improve fermentation quality, enhance lactic acid production, inhibit undesirable microorganisms, and reduce antibiotic resistance genes, offering a sustainable and natural strategy for high-quality silage production.

Astragali Radix (*Stragalus membranaceus* L., AR) is a renowned traditional Chinese herbal medicine originating from traditional Chinese medicine (TCM), holding a prominent position in TCM due to its wide-ranging therapeutic effects and health-promoting properties [17]. Astragali Radix primarily contains bioactive compounds such as Astragalus polysaccharides (APS), Astragaloside IV (AS-IV), and Calycosin, which can significantly influence microbial growth [18,19,20]. As a plant of significant medicinal value, Inulae Radix (*Inula helenium* L., IR) has demonstrated broad applications in both traditional Chinese medicine and modern medicine. Its roots are rich in sesquiterpene lactones and essential oils, including dehydrocostus lactone, costunolide, terpinene, and borneol. The antioxidant capacity of *Inula helenium* is attributed to its polyphenolic and flavonoid constituents, which exhibit notable antibacterial and antimicrobial activities [21,22]. *Inula helenium* and *Astragalus membranaceus* have long been used in traditional Chinese medicine and are highly valued for their immunomodulatory, anti-inflammatory, antioxidant, and digestive-promoting properties.

While most existing studies have focused on using Chinese herbal medicine residues as silage additives, there is no prior report on the direct application of AR and IR in barley ensiling. This study therefore represents a novel attempt to harness the bioactive compounds of these two herbs—aiming to suppress undesirable microorganisms and promote lactic acid bacteria fermentation—specifically in barley silage. Despite these promising results in crops such as corn and alfalfa, the potential of Chinese herbal medicine additives in barley ensiling is poorly understood. This study aims to bridge this gap by investigating the effects of *Astragalus membranaceus* L. (Astragali Radix, AR) and *Inula helenium* L. (Inulae Radix, IR) on barley silage. Key parameters including fermentation characteristics, nutrient retention, and overall feed quality were systematically evaluated to inform strategies for improved forage conservation.

## 2. Materials and Method

### 2.1. Silage Production and Sampling

Barley was cultivated at the Experimental Station of the Inner Mongolia Academy of Agricultural and Animal Husbandry Sciences. Astragali Radix and Inulae Radix were purchased from Tongrentang (Tongrentang Co., Ltd, Beijing, China). Barley plants were harvested at the milk-ripe stage by cutting it about 10 cm above the ground level, and then chopped it to a theoretical length of 1 cm; the nutrient composition of the raw materials is presented in Table 1. The above two herbal medicines were dried at 40 °C until constant weight was achieved, then ground to 100 mesh using a herb-specific grinder (CHIGO Industry Co., Ltd, Guangdong, China), and stored for subsequent use. After thorough mixing, each treatment was weighed into 300 g portions, with three independent biological replicates prepared (three separate silo bags, *n* = 3) and treated as follows: (CK) non-additive; (AR-1%) applied with 1% (*w*/*w*) Astragali Radix; (AR-2%) 2% (*w*/*w*) Astragali Radix; (IR-1%) 1% (*w*/*w*) Inulae Radix; (IR-2%) 2% (*w*/*w*) Inulae Radix. No additional microbial inoculants were added. The total number of samples across all treatment groups is 15 (5 treatments × 3 replicates). Each treatment of silage was packed into a polyethylene plastic bag and sealed with a vacuum sealer (Baijie Industry Co., Ltd., Zhejiang, China). All silage was stored in room temperature for 75 days.

**Table 1:** The nutritional components of raw barley materials.

DM (%)	CP/%DM	NDF/%DM	ADF/%DM	WSC/%DM
26.40 ± 0.10	13.40 ± 0.14	48.17 ± 0.18	25.13 ± 0.18	5.19 ± 0.12

### 2.2. Chemical Composition and Fermentation Characteristic Analysis

After ensiling for 75 days, all silage was dried by a thermostatic dryer (BOXUN Industry Co., Ltd, Shanghai, China) at 65 °C until constant weight and ground to 1 mm using a mill (Shijiazhuang Chenxing Industry Co., Ltd, Hebei, China) for detecting dry matter, neutral detergent fiber (NDF), and acid detergent fiber (ADF) content. The analysis of NDF and ADF was measured using the ANKOM method [23]. Ammonia nitrogen (NH_3_-N) and total nitrogen (TN) concentrations of silage were determined using the phenol-hypochlorite method [24]. Crude protein and ash content were measured by the Kjeldahl method [25]. Crude ash was determined using the AOAC method. The water-soluble carbohydrate and starch content of dried silage sample was detected using phenol-sulfuric acid method according to the previous research [26]. Fresh silage (20 g) was homogenized with 180 mL sterile water. The mixture was then centrifuged at 5000 r/min for 10 min. Subsequently, the pH of the resulting supernatant was measured using pH meter (Mettler-Toledo International Inc, Greifensee, Switzerland). For the analysis of organic acids, including lactic acid (LA), acetic acid (AA), propionic acid (PA), and butyric acid (BA), the supernatant was filtered through a 0.22 μm nylon membrane filter (Tianjin Jinteng Experiment Equipment Co., Ltd, Tianjin, China). The filtered extracts were analyzed using a high-performance liquid chromatography (HPLC) system equipped with PDA detector (E2695, Waters Corporation, Milford, MA, USA)set at 210 nm. Separation was achieved on a KC-811 column (2.1 × 150 nm, 3.5 μm; Shimadzu Co., Kyoto, Japan) maintained at 50 °C. The mobile phase was 3 mmol/L perchloric acid aqueous solution. The flow rate was 1 mL/min and the injection volume was 10 μL.

### 2.3. Microbial Analysis

After opening the sample bags, the contents of each bag were thoroughly homogenized. Nucleic acid extraction was performed using the Soil DNA Extraction Kit (D5635-02) (Omega Bio-Tek, Norcross, GA, USA). The extracted DNA was first evaluated for fragment size by 0.8% agarose gel electrophoresis, followed by concentration determination using a Nanodrop spectrophotometer (Thermo Fisher Scientific, Waltham, MA, USA).. Targeting the bacterial 16S rRNA gene V3-V4 region (~468 bp), PCR amplification was carried out with the primers 338F (5′-barcode+ACTCCTACGGGAGGCAGCA-3′) and 806R (5′-GGACTACHVGGGTWTCTAAT-3′). The amplification products were used to construct libraries with the TruSeq Nano DNA LT Library Prep Kit, and the library concentrations were measured using a Bio Tek Flx800 microplate reader (Agilent Technologies, Santa Clara, CA, USA).

### 2.4. Statistical Analysis

Data were initially organized and subjected to preliminary analysis using Microsoft Excel 2016. For parametric data—including chemical composition and fermentation quality—statistical analyses were performed using SPSS version 21.0 (IBM Corp., Armonk, NY, USA) based on a general linear model, with treatment means compared via Duncan’s multiple range test. Microbial community data were analyzed through BMKCloud (www.biocloud.net, accessed on 30 October 2025), where group differences in alpha diversity and taxonomic abundance were assessed using Tukey’s honestly significant difference test. All results are presented as mean ± standard deviation (SD). Differences were considered statistically significant at *p* < 0.05.

## 3. Results

### 3.1. Effect of AR and IR on Fermentation Quality

**Figure 1:**
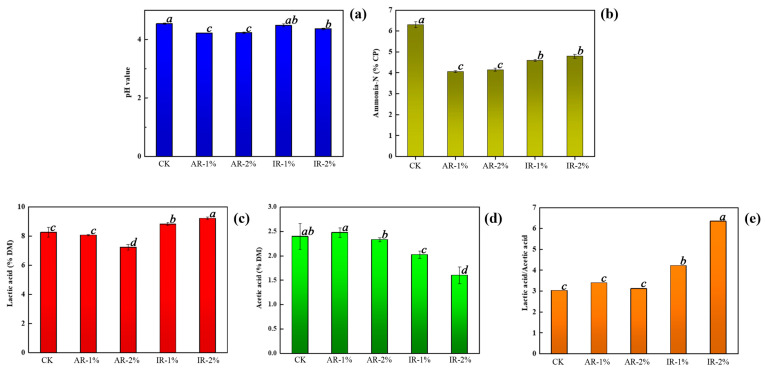
Fermentation characteristics in barley silage during ensiling. (**a**) pH; (**b**) Ammonia-N content; (**c**) Lactic acid content; (**d**) Acetic content; (**e**) Lactic acid/acetic acid. CK, barley silage with non-additive; AR-1%, barley silage with 1% Astragali Radix additive; AR-2%, barley silage with 2% Astragali Radix additive; IR-1%, barley silage with 1% Inulae Radix additive; IR-2%, barley silage with 2% Inulae Radix additive. Data within the same row labeled with the same lowercase letter or without a letter indicate no significant difference (*p* > 0.05), whereas different lowercase letters indicate significant differences (*p* < 0.05). The same applies to the following tables and figures.

The fermentation parameters of barley silage treated with different additives are presented in Figure 1. The application of all additives significantly influenced the fermentation profile, including pH, NH_3_-N (% of CP), lactic acid (LA), and acetic acid (AA) concentrations (% of DM), and the LA/AA ratio. The pH values were significantly lower in all treatment groups than in the CK, with the AR groups achieving the most pronounced reduction. The NH_3_-N content was significantly reduced in all additive-treated groups (*p* < 0.05), with the lowest values recorded in the AR groups. Compared with the CK and AR treatment, IR addition notably enhanced lactic acid production. The highest LA content was observed in the 2% IR group, which was significantly greater than all other groups (*p* < 0.05). In contrast, both AR groups showed a reduction in LA concentration relative to the control. Furthermore, the LA/AA ratio was highest in the 2% IR treatment, indicating a more homolactic fermentation pattern, which is generally associated with higher aerobic stability and better fermentation quality.

### 3.2. Effect of AR and IR on Chemical Composition of Barley Silage

The chemical composition of barley silage treated by different additives is presented in Table 2. Both AR and IR additives significantly influenced the contents of crude protein (CP), neutral detergent fiber (NDF), acid detergent fiber (ADF), starch, water-soluble carbohydrates (WSC), and crude ash (ash) after 75 days of ensiling. The results indicated that all additive-treated groups had significantly higher dry matter (DM) content compared to the CK (*p* < 0.05), with the 2% IR group exhibiting the highest DM value. In terms of CP, the AR treatments did not lead to significant changes relative to the control. In contrast, both the 1% and 2% IR treatments resulted in significantly lower CP contents. The NDF and ADF contents decreased significantly in all additive-treated groups compared to the CK (*p* < 0.05). Similarly, ash content was also reduced across all treatment groups. Starch content increased markedly with additive application, with the 1% IR group showing the highest value, followed by the 2% IR group. All treated silage exhibited significantly higher WSC content than the control (*p* < 0.05), and the 1% IR treatment resulted in the highest WSC concentration among all groups.

### 3.3. Microbial Diversity and Structural Differences

**Figure 2:**
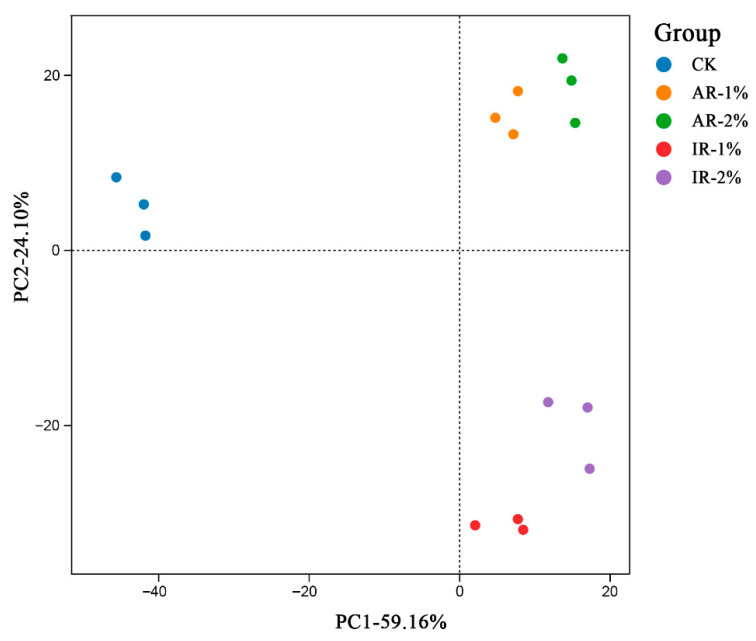
PCA (principal component analysis) based on OTU level of barley silage by NGS sequencing.

Principal component analysis (PCA) revealed clear separation in the overall microbial community structure (OTU level) among the treatment groups (Figure 2). The first two principal components (PC1 and PC2) accounted for 59.16% and 24.10% of the total variation, respectively. The control group (CK) was distinctly separated from the groups treated with Astragali Radix (AR-1%, AR-2%) and Inulae Radix (IR-1%, IR-2%) along PC1, which captured the majority of the variance. Furthermore, the clusters for AR and IR treatments showed separation from each other, implying that the type and concentration of the additive induced specific and distinguishable shifts in the bacterial community.

**Figure 3:**
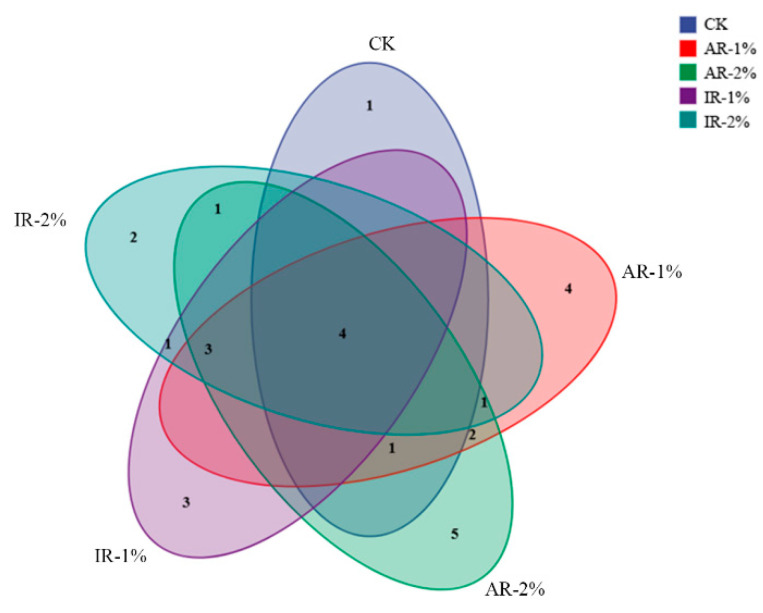
Venn diagram showing the coincidence of species among the groups (relative abundance > 1%).

The Venn diagram (Figure 3) illustrated the distribution of shared and unique bacterial species (relative abundance > 1%) among the different treatment groups after ensiling. A core microbiome, consisting of species common to all groups, was identified, underscoring a stable microbial foundation in barley silage. However, the number of unique species varied notably among the treatments. The CK harbored a distinct set of four unique species. In contrast, the additive-treated groups exhibited variations in their unique species counts: the AR-2% and IR-2% groups contained 2 and 4 unique species, respectively, while the AR-1% and IR-1% groups contained 1 and 3.

### 3.4. Microbial Community Composition of Barley Silage

**Figure 4:**
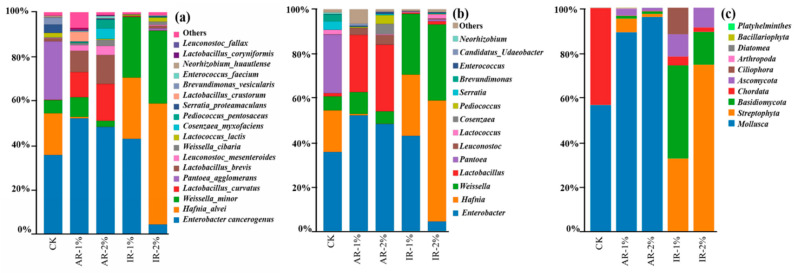
The relative top tenth abundance of microbial community at species level (**a**), genus level (**b**), and phylum level (**c**) of barley silage after 75 days of ensiling.

The microbial community structure of barley silage, analyzed at the species, genus, and phylum levels following 75 days of ensiling, is shown in Figure 4. The composition was significantly influenced by the application of different additives.

At the species level (Figure 4a), the CK was predominantly composed of *Enterobacter cancerogenus*, *Pantoea agglomerans*, *Hafnia alvei*, and *Weissella minor*. Notably, the relative abundance of *Enterobacter cancerogenus* was significantly reduced in the IR-2% group, whereas it increased in the AR-1%, AR-2%, and IR-1% groups compared to the control. The abundance of *Hafnia alvei* decreased in AR-treated silages but increased in IR-treated groups. Conversely, *Enterobacter cancerogenus* was significantly reduced only in IR-2% treatment. Both AR treatments markedly promoted the dominance of lactic acid bacteria (LAB), primarily through the enrichment of *Lactobacillus curvatus* and *Lactobacillus brevis*.

At the genus level (Figure 4b), the control silage exhibited high abundance of *Weissella*, *Leuconostoc*, and *Hafnia*. Additive applications substantially suppressed these genera while enhancing the proliferation of *Lactobacillus*. This structural shift was most pronounced in IR-treated silages, where *Lactobacillus* emerged as the predominant genus.

At the phylum level (Figure 4c), the addition of AR significantly inhibited the growth of *Mollusca* compared to the CK (*p* < 0.05). In contrast, AR-2% treatment suppressed the growth of *Streptophyta* compared to the AR-1% treatment.

The addition of IR completely inhibited the growth of *Mollusca* and promoted the growth of *Streptophyta*, *Basidiomycota*, and *Ascomycota*. The IR-1% treatment resulted in a significantly stronger promotion of *Basidiomycota* growth than IR-2% treatment, whereas its promoting effect on *Streptophyta* was significantly lower than that of IR-2% treatment.

### 3.5. Correlation Analysis Between Microbial Taxa and Silage Characteristics

**Figure 5:**
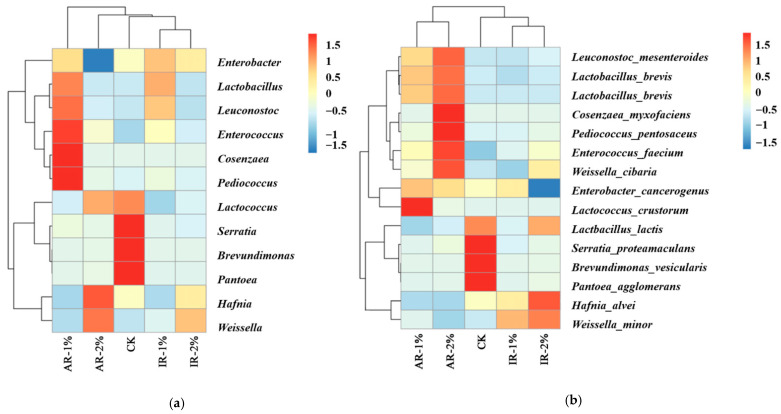
Heatmap of the relative abundance of the top 15 microbial genera (**a**) and species (**b**).

As shown in Figure 5, the heatmap displays the relative abundance of the top fifteen microbial genera (a) and species (b). The results indicate that in the CK treatment group, the abundances of the genera *Serratia*, *Brevundimonas*, and *Pantoea* were the highest, corresponding to the species *Serratia proteamaculans*, *Brevundimonas vesicularis*, and *Pantoea agglomerans* at the species level. The species *Lactbacillus lactis* under the genus *Lactococcus* had an abundance greater than 1, while the abundance of other species was less than 0.5. In the AR-1% treatment, the genera *Cosenzaea* and *Pediococcus* had the highest abundances, while in the AR-2% treatment, the species *Cosenzaea myxofaciens* and *Pediococcus pentosaceus* had the highest abundances. In the treatments with added IR, the species abundances were relatively uniform compared to other treatments. Specifically, in the IR-1% treatment, the abundances of the genus Lactobacillus and the species *Weissella* minor were the highest, whereas in the IR-2% treatment, the abundances of the genus *Weissella* and the species *Hafnia alvei* were the highest. This difference is mainly due to the higher abundances of Lactobacillus brevis and *Lactococcus lactis* within the *Lactobacillus* genus in the IR-1% treatment, although both were lower than *Weissella* minor. In the IR-2% treatment, the species *Weissella* *cibaria* and *Weissella minor* within the *Weissella* were present, but both were lower in abundance than *Hafnia alvei*.

### 3.6. Correlation Analysis Between Silage Characteristics and Microbial Taxa

**Figure 6:**
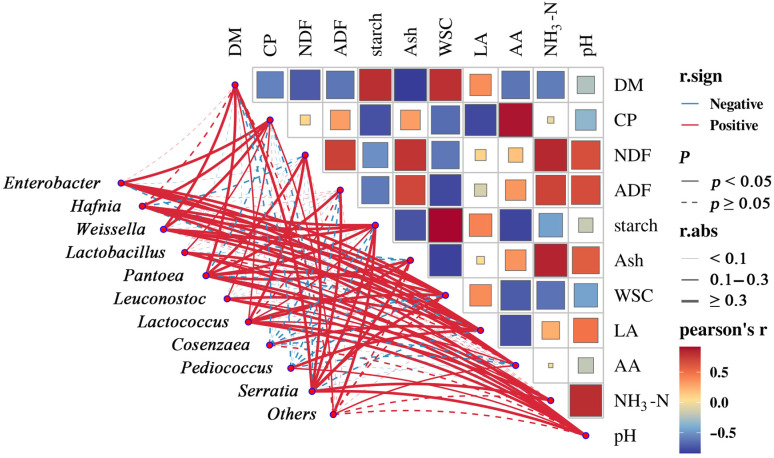
Correlations between silage characteristics and the abundance of microbial genera.

To elucidate the interrelationships among fermentation parameters, nutrient composition, and the microbial community, Spearman correlation analysis was performed and shown in Figure 6. The results revealed significant correlations (|r| > 0.8, *p* < 0.05) among key silage characteristics. Specifically, starch, ash, and WSC showed strong correlations DM; AA with crude protein CP; NDF with NH_3_-N; starch with AA; and WSC and NH_3_-N with ash. Additionally, pH was strongly correlated with NH_3_-N. Among these, ash was negatively correlated with DM, starch with AA, and WSC with ash.

At the microbial level, several genera exhibited significant correlations (|r| > 0.5, *p* < 0.05) with silage traits. *Enterobacter* correlated positively with WSC, CP, and AA. *Hafnia* showed positive correlations with LA, CP, and AA. *Lactobacillus* and *Lactococcus*, as primary lactic acid bacteria, were positively associated with LA and pH, underscoring their central role in acid production and fermentation pH dynamics. *Pediococcus* was positively correlated with LA, indicating its focused role in homolactic fermentation. *Pantoea* and *Serratia* demonstrated significant positive correlations with multiple structural and nutritional components, including ADF, DM, NDF, WSC, ash, and NH_3_-N, suggesting their potential involvement in carbohydrate metabolism and fiber decomposition. Furthermore, *Lactococcus*, *Pantoea*, and *Serratia* showed positive correlations with NH_3_-N, implying a potential link to protein degradation or nitrogen metabolism pathways.

## 4. Discussion

Ensiling is a microbial-driven process where the rapid establishment of an acidic environment is crucial for inhibiting spoilage microorganisms and preserving nutrients [27]. In line with this principle, the significant reduction in pH and increase in lactic acid (LA) content across all additive-treated groups indicates that both AR and IR additives effectively enhanced the fermentation process of barley silage. The most pronounced pH reduction in the AR groups was likely a direct result of rapid acidification, presumably driven by enriched *Lactobacillus* populations, as evidenced by microbial analysis. This mechanism is consistent with previous findings that plant-derived compounds can stimulate LAB activity, thereby accelerating pH decline [28,29]. Huang et al. reported that total flavonoids from *Taraxacum mongolicum* have been shown to improve fermentation quality and nutrient preservation in silage, likely by influencing microbial communities [28]. Similarly, citric acid, when combined with *Lactobacillus plantarum*, alters the bacterial community of king grass silage, reducing bacterial diversity while enhancing the abundance of desirable strains and improving fermentation quality [29].

Notably, the highest LA concentration and LA/AA ratio observed with 2% IR barley silage indicated a promotion of homolactic fermentation, which is crucial for preserving fermentable substrates and minimizing nutrient losses in various fermented products like alfalfa silage [30,31,32]. The increased LA production at 2% IR suggests that this herbal additive effectively modulates fermentation pathways towards a more desirable profile. Although the IR-2% treatment exhibited the highest lactic acid (LA) concentration, its pH was not the lowest, likely because the acetic acid (AA) content in this treatment was lower than in the other treatments. In contrast, the reduction in LA content in AR groups relative to the control, despite a lower pH, suggests a possible shift in microbial metabolism or the presence of antimicrobial compounds affecting specific LAB strains [30].

The pronounced increase in starch and water-soluble carbohydrate (WSC) content in all additive-treated silage, particularly with IR, aligns with prior findings that a rapid pH decline helps preserve non-fiber carbohydrates [33]. Feng et al. [34] reported that the fermentation quality and lactic acid content of hybrid *Pennisetum* were significantly improved by the addition of lactic acid bacteria or organic acids during ensiling. Concurrently, the reduction in neutral detergent fiber (NDF) and acid detergent fiber (ADF) across all treatment groups suggests improved fiber digestibility, potentially mediated by acid hydrolysis or the indirect suppression of fibrolytic microbes [34,35]. Although all additive groups showed a lower crude protein (CP) content, their significantly lower ammonia-nitrogen (NH_3_-N) levels demonstrate an effective suppression of proteolysis. This phenomenon, consistent with the report by Li et al., demonstrated that the addition of clove, mint, and purple perilla residues significantly reduced the ammonia-nitrogen/total nitrogen ratio and non-protein nitrogen content in paper mulberry silage, indicating effective suppression of proteolysis and deamination [36].

The present study demonstrated that the use of herbal additives, Astragali Radix and Inulae Radix, significantly altered the microbial community structure in barley silage. The principal component analysis (PCA) revealed a clear separation between the control and all additive-treated groups of barley silage, indicating that both the type and concentration of herbal additives were decisive factors in shaping the bacterial community. This finding is consistent with previous findings that herbal residues can selectively inhibit or promote specific microbial populations of paper mulberry silage [36]. The Venn diagram provided further evidence of additive-induced microbial selection. The control group maintained a distinct suite of unique species, predominantly epiphytic or opportunistic bacteria originating from the field environment. In contrast, the reduction in unique species counts in AR-1% and IR-1% group indicated a structural simplification of the bacterial community [37]. Li et al. reported *Perilla frutescens* was effective in combating the spoilage of oat silage for feed, which is similar to the mechanism of action of Astragali Radix and Inulae Radix in the present study [38]. The biochemical mechanism underlying this selective inhibitory effect may be related to bioactive compounds present in plant additives, such as flavonoids, polyphenols, or alkaloids. These compounds can suppress non-target microbes by disrupting cell walls, membranes, enzymes, or DNA, while being less toxic or even beneficial to lactic acid bacteria, giving them a competitive edge [36,39].

The dynamics of microbial communities during barley silage ensiling are significantly influenced by the application of different additives. Both herbal additives, especially at the 2% application rate, markedly promoted the dominance of the beneficial genus *Lactobacillus*. At the species level, this was primarily driven by the enrichment of *Lactobacillus curvatus* and *Lactobacillus brevis*. These species were known homofermentative or facultatively heterofermentative LAB, capable of efficiently converting water-soluble carbohydrates into lactic acid, which explains the rapid pH drop and high LA production observed in these groups [40].

In the CK, due to the absence of external intervention, fermentation relied primarily on the indigenous microbial community present in the original forage environment. The results showed that the dominant genera were typical environmental bacteria, such as *Serratia* and *Pantoea*. The AR-treated groups likely contained specific bioactive compounds that provided favorable resources for *Cosenzaea-* and *Pediococcus*-related microbes. For instance, *Pediococcus* is a typical lactic acid bacterium (LAB) genus that often dominates under acidic conditions; its increased abundance in AR treatments is presumably linked to the fermentation characteristics induced by this additive. Some studies have indicated that Astragalus polysaccharides can directly promote the proliferation of *Pediococcus*, thereby optimizing the microbial environment—similar mechanisms may operate in silage systems [41,42]. In contrast, IR groups exhibited a clear advantage of lactic acid bacteria, particularly *Lactobacillus brevis* and *Lactococcus lactis,* as well as *Weissella*-related species. This suggests that IR may contain components that enhance lactic acid metabolic activity, such as compounds capable of rapidly lowering pH and shifting the environment toward conditions favorable for LAB proliferation. Notably, in the IR-2% treatment, *Hafnia alvei* became the dominant species, which may be attributed to the higher dosage of IR altering substrate utilization patterns or suppressing certain competing microbial taxa [43,44].

The observed negative correlation between DM and ash content can be attributed to a dilution effect, where a higher proportion of organic matter effectively reduces the relative percentage of inorganic minerals [45]. The positive correlation between WSC and DM is expected, as soluble sugars constitute a direct component of the dry matter [46]. The relationship between NDF and NH_3_-N is complex and context-dependent. While not universally fixed, under specific ensiling conditions (e.g., with additives), enhanced protein degradation (reflected by higher NH_3_-N) can sometimes coincide with reduced NDF, and ample WSC can promote efficient lactic fermentation, which may suppress proteolysis and thus lower NH_3_-N concentration [47,48]. The significant negative correlation between WSC and ash aligns with findings in modern fermented feed research, where increased fermentable carbohydrates reduce the relative ash proportion [35].

The strong positive correlations of *Lactobacillus* and *Lactococcus* with LA confirm their canonical function as core lactic acid producers, directly driving pH reduction for stable preservation [49,50]. The association of *Enterobacter* with WSC and CP suggests its possible role in sugar and nitrogen metabolism. *Pediococcus* appears to specialize in LA production, representing a more targeted metabolic function [43]. The broad positive correlations of *Pantoea* and *Serratia* with fibrous components (ADF, NDF) and WSC indicate a potential, though not yet fully elucidated, role in carbohydrate utilization, warranting further investigation. Their positive link to NH_3_-N, shared with *Lactococcus*, points to a possible involvement in nitrogenous compound transformation during ensiling [51,52].

## 5. Conclusions

Herbal additives can significantly enhance the fermentation quality and nutritional value of barley silage by selectively modulating the microbial community. IR, particularly at a 2% application rate, proved to be highly effective in promoting a homolactic fermentation type, as evidenced by the highest lactic acid content and LA/AA ratio, leading to superior aerobic stability. Both additives successfully suppressed spoilage bacteria and enriched beneficial *Lactobacillus* populations, thereby accelerating acidification and reducing protein degradation. The correlation and functional analyses provided deeper insights into the mechanistic relationships between key microbial taxa and silage quality parameters. These findings demonstrate the great potential of Astragali Radix and Inulae Radix as natural, effective, and sustainable microbial regulators for silage preparation, offering a viable strategy to improve silage production, especially in regions seeking alternatives to commercial inoculants.

## Figures and Tables

**Table 2:** Nutritive components of barley silage after ensiling.

Items	CK	AR-1%	AR-2%	IR-1%	IR-2%	*p* Value
DM (%)	25.02 ± 0.11 ^b^	26.18 ± 0.23 ^a^	26.34 ± 0.36 ^a^	27.07 ± 0.62 ^a^	27.12 ± 0.68 ^a^	<0.01
CP/%DM	12.78 ± 0.11 ^a^	12.94 ± 0.19 ^a^	12.84 ± 0.19 ^a^	11.86 ± 0.05 ^c^	12.20 ± 0.24 ^b^	0.034
NDF/%DM	49.67 ± 0.74 ^a^	46.55 ± 1.16 ^b^	46.34 ± 0.71 ^b^	47.14 ± 0.96 ^b^	46.70 ± 1.05 ^b^	<0.01
ADF/%DM	26.10 ± 0.44 ^a^	24.51 ± 0.51 ^b^	24.60 ± 0.61 ^b^	24.10 ± 0.38 ^b^	25.01 ± 0.58 ^b^	0.047
Starch/%DM	0.26 ± 0.03 ^e^	0.77 ± 0.05 ^d^	1.93 ± 0.10 ^c^	2.86 ± 0.07 ^a^	2.14 ± 0.07 ^b^	<0.01
Ash/%DM	5.80 ± 0.06 ^a^	5.38 ± 0.04 ^b^	5.23 ± 0.13 ^b^	5.29 ± 0.06 ^b^	5.29 ± 0.08 ^b^	<0.01
WSC/%DM	1.71 ± 0.01 ^d^	2.89 ± 0.06 ^c^	3.18 ± 0.04 ^b^	4.15 ± 0.07 ^a^	3.13 ± 0.11 ^b^	<0.01

^a–e^ Means in the same row with different subscripts differ significantly (*p* < 0.05). DM, dry matter; CP, crude protein; NDF, neutral detergent fiber; ADF, acid detergent fiber; WSC, water soluble carbohydrates.

## Data Availability

The original contributions presented in this study are included in the article. Further inquiries can be directed to the corresponding author.
